# *In Situ* Measurement of Adhesion for Multimetallic Nanoparticles

**DOI:** 10.1021/acs.nanolett.5c00076

**Published:** 2025-04-15

**Authors:** Andrew Baker, Sai Bharadwaj Vishnubhotla, Sanjana Karpe, Yahui Yang, Götz Veser, Tevis D. B. Jacobs

**Affiliations:** † Department of Mechanical Engineering and Materials Science, University of Pittsburgh, Pittsburgh, Pennsylvania 15260, United States of America; ‡ Department of Chemical and Petroleum Engineering, University of Pittsburgh, Pittsburgh, Pennsylvania 15260, United States of America; § Center for Integrated Nanotechnologies, Sandia National Laboratories, Albuquerque, New Mexico 87123, United States of America

**Keywords:** Nanoparticles, Adhesion, *In situ* TEM, Bimetallic nanoparticles, Metal/oxide interface, Multimetallic

## Abstract

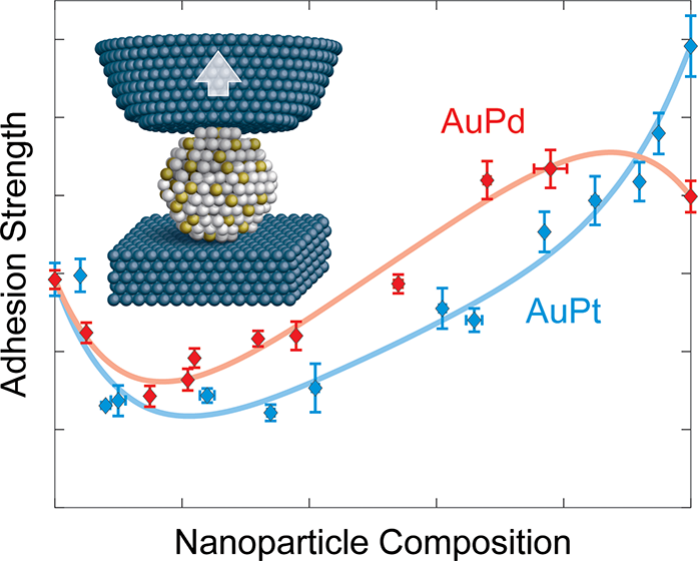

The adhesion of nanoparticles to their supports is key to their performance and stability. However, scientific advances in this area have been hampered by the difficulty of experimentally probing adhesion. To date, only a single technique has been developed that can *directly* measure nanoparticle adhesion, and this technique is inherently limited to monometallic systems. We present a versatile technique for the direct measurement of adhesion for bimetallic nanoparticle systems. This technique combines the spatial resolution of transmission electron microscopy with the force resolution of an atomic force microscope to probe individual, well-characterized nanoparticles. A first study of supported bimetallic nanoparticles provides new insights into the complex impact of alloying on nanoparticle adhesion, explained by charge transfer between constituent metals. The new experimental technique is readily extensible to study other multimetallic nanoparticle systems, including the effects of particle size, shape, and orientation, thus enabling advances in our understanding of nanoparticle physics.

Metal–support interactions encompass a range of physical mechanisms—including chemical bonding, electrostatic forces, and van der Waals interactions—which collectively govern the adhesion of metal nanoparticles to a support surface and play a pivotal role in shaping the properties and performance of functional nanomaterials.^1,2^ Key technologies, such as power generation and storage,^3^ catalysis,^4^ and sensing^5^ rely on nanoparticles, typically supported on an oxide surface. In these systems, metal–support interactions are critical to controlling chemical and physical properties and to preventing nanoparticle aggregation and hence loss of functional properties.^6−9^

However, despite the crucial role of nanoparticle adhesion, scientific advances have been hampered by the difficulty of probing this adhesion experimentally. The most common way to measure adhesion is using particle shape measurements,^10^ but this technique relies on an assumption that the particle is in its equilibrium shape. The only *direct* measurement of nanoparticle adhesion is single-crystal adsorption calorimetry.^11^ While this pioneering technique has revealed the effects of chemical potential,^12^ nanoparticle size,^13^ oxophililicity,^14^ and oxidation state of the oxide support^15^ on adhesion, its use is inherently limited to monometallic particles. Direct measurement of the adhesion of nanoparticles containing two or more metals, such as bimetallic nanoparticles which offer a wealth of emergent properties,^16−19^ has been elusive thus far. The physics of nanoparticle adhesion for multimetallic nanoparticles hence remains poorly understood, creating a critical need for an experimental technique to investigate this important class of materials.

Here we report a novel technique that enables direct measurement of nanoparticle adhesion. The technique couples the use of *in situ* transmission electron microscopy (TEM) for characterization of the size, shape, orientation, and composition of the materials (Figure 1) with the simultaneous, high-resolution measurement of adhesion force *F*_adh_ and particle/oxide contact area *A*_cont_ (for more details, please see Figure S1 and Movie S1). This technique enables the measurement of the force per unit area required to separate the contact. Under the present conditions, this provides a direct measurement of the adhesion strength σ_adh_ of the interface.^20^ (Please see Supplementary Note 1 for explicit discussion of how to determine interfacial properties.) As discussed there, the adhesion strength is an intrinsic property of the interface and is proportional to adhesion energy *E*_adh_.

**Figure 1:**
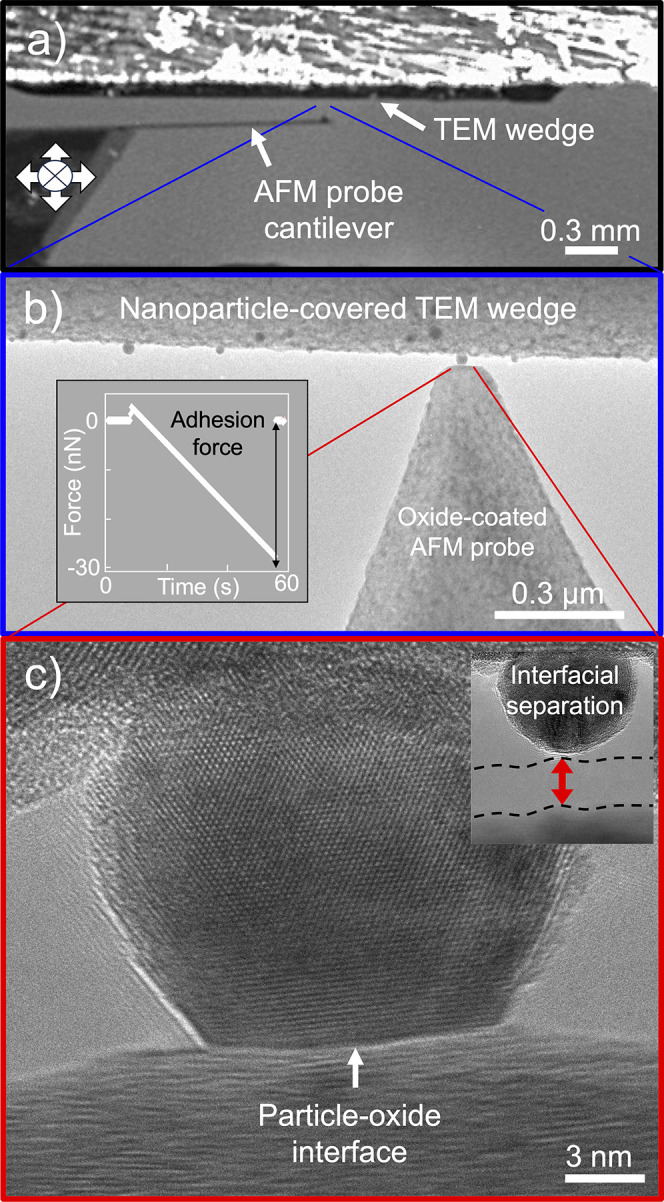
***In situ* measurement of nanoparticle-support interactions**. (a) An *in situ* nanomechanical test platform brings metal nanoparticles, deposited on a wedge-shaped substrate, into contact with an oxide surface, deposited on a cantilever load sensor (see Methods). (b) Adhesion tests are performed inside a transmission electron microscope, enabling the targeted selection of individual particles for investigation. The real-time force is measured (inset) throughout each test, enabling the extraction of the adhesive force. A full video of a test can be found in the Supporting Information (Movie S1). (c) Real-time high-resolution measurements enable determination of the size and shape of the nanoparticle along with the separation force of the interface (top-right inset shows the particle and cantilever tip after separation).

We validated the novel technique using six different combinations of monometallic nanoparticles and oxide support materials in two ways: first, the adhesion strength was measured for a single metal (Au) on three different oxides (CeO_2_, TiO_2_, and MgO); this was followed by measurements of four different metal nanoparticles (Pt, Pd, Ag, Au) on a single support, CeO_2_. The metal nanoparticles were intentionally chosen to be sufficiently large (15–40 nm) to avoid size-dependent adhesion effects that are expected below approximately 6 nm.^14,24^ For nanoparticles of the same metal (Au) adhered to three different oxides (Figure 2a), the adhesion strength decreases in the order CeO_2_ > TiO_2_ > MgO (σ_adh_ = 584 ± 22, 303 ± 27, and 102 ± 11 MPa, respectively). For the different metals on ceria (Figure 2b), results show decreasing adhesion strength in the following order: Pt > Pd > Au ≈ Ag (σ_adh_ = 1183 ± 78, 797 ± 41, 584 ± 22, and 572 ± 19 MPa, respectively). These results of adhesion strength are in close agreement (*R*^2^ > 0.9) with trends in adhesion energy reported in prior literature,^14^ with subsequent updated values,^22^ thus validating the method against the current state-of-the-art technique for adhesion measurement (Figure 2c; for a full discussion of the comparison, see Supplementary Note 2).

**Figure 2:**
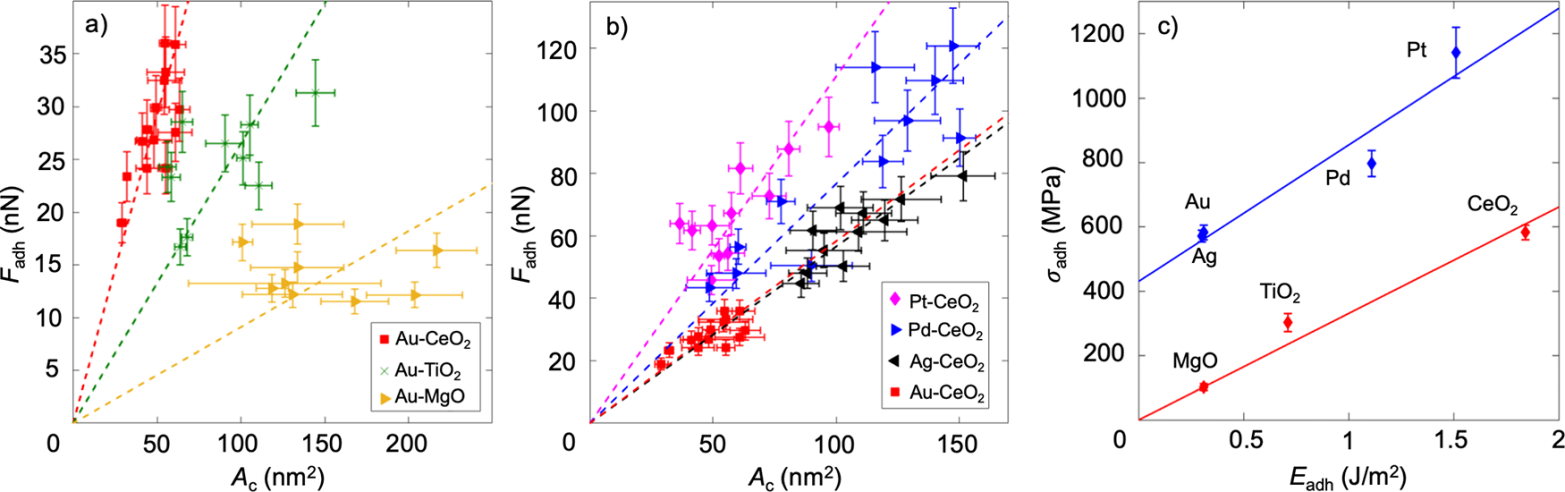
**Adhesion measurements for six different nanoparticle-support systems serve to validate the technique**. Adhesion force and contact area were measured for (a) gold nanoparticles on CeO_2_, TiO_2_, and MgO supports, as well as for (b) nanoparticles of platinum, palladium, silver, and gold on CeO_2_ supports. This enables the determination of adhesion strength σ_adh_ (the slope of the dashed lines in (a) and (b)), which is characteristic of each material system. (c) The novel technique is validated by confirming proportionality of the obtained adhesion strength to previously reported trends in adhesion energy *E*_adh_.^14,22^ As described in the main text, the red data compares the measurements from panel (a) to prior measurements on identical materials; the blue data compares the measurements from panel (b) to the same metals on a different substrate (MgO), which explains the vertical offset. The solid lines are linear fits, and the *R*^2^ values are 0.92 and 0.97, for the blue and red lines, respectively.

While the current results can be compared against prior literature, inherent differences in the techniques prevent a straightforward comparison of absolute values. The present force-based technique directly determines the maximum negative force required to separate the interface; when normalized by contact area, this yields the adhesion strength, σ_adh_. In contrast, single-crystal adsorption calorimetry is an energy-based technique that directly determines the energy change upon formation of the interface; when normalized by contact area, this yields the adhesion energy *E*_adh_. These two properties of the interface scale proportionally with one another; therefore the test for validation is direct proportionality (i.e., linear correlation) between the measured adhesion strengths and previously reported adhesion energies. These results are shown in Figure 2c. The results show that, for gold, which has been measured previously on MgO, TiO_2_, and CeO_2_, we obtain a very close correlation between our results and prior results with an R^2^ value of 0.97. For various metals on CeO_2_, we ran into the problem that these systems have never been measured before. Therefore, we compared these metals (Ag, Au, Pd, Pt) against prior measurements on a different substrate, MgO, under the hypothesis that the trends between the different metals will be identical, with only a (constant) offset in absolute values. Indeed, these results show close linearity as well, with an *R*^2^ value of 0.92. This proportionality between currently measured results and prior literature hence validates the present method against the current state-of-the-art technique for adhesion measurement (For a full discussion of the comparison, see Supplementary Note 2.)

Following validation of the technique, we investigated the adhesion of bimetallic systems, specifically AuPt and AuPd, chosen because of their similarity as noble metals and their tendency to yield synergistic effects in catalysis.^17,25−27^ Particles were synthesized in the composition range between 0% and 100% Au using straightforward wet impregnation (see Methods). For each bimetallic system (AuPt and AuPd), more than 100 *in situ* adhesion tests were performed on ten or more different compositions across the composition spectrum. All *in situ* adhesion tests were performed with CeO_2_ as the support material due to its technological relevance.^28^ For every nanoparticle tested, *in situ* energy dispersive X-ray spectroscopy (EDS) was used to assess the composition and spatial distribution of each metal in the system (see Figure S3 for more detail). These direct measurements of bimetallic nanoparticle adhesion reveal strongly non-monotonic trends in adhesion with varying composition, with qualitatively different behavior for AuPd (S-shaped curve; Figure 3a) and AuPt (concave curve; Figure 3b).

**Figure 3:**
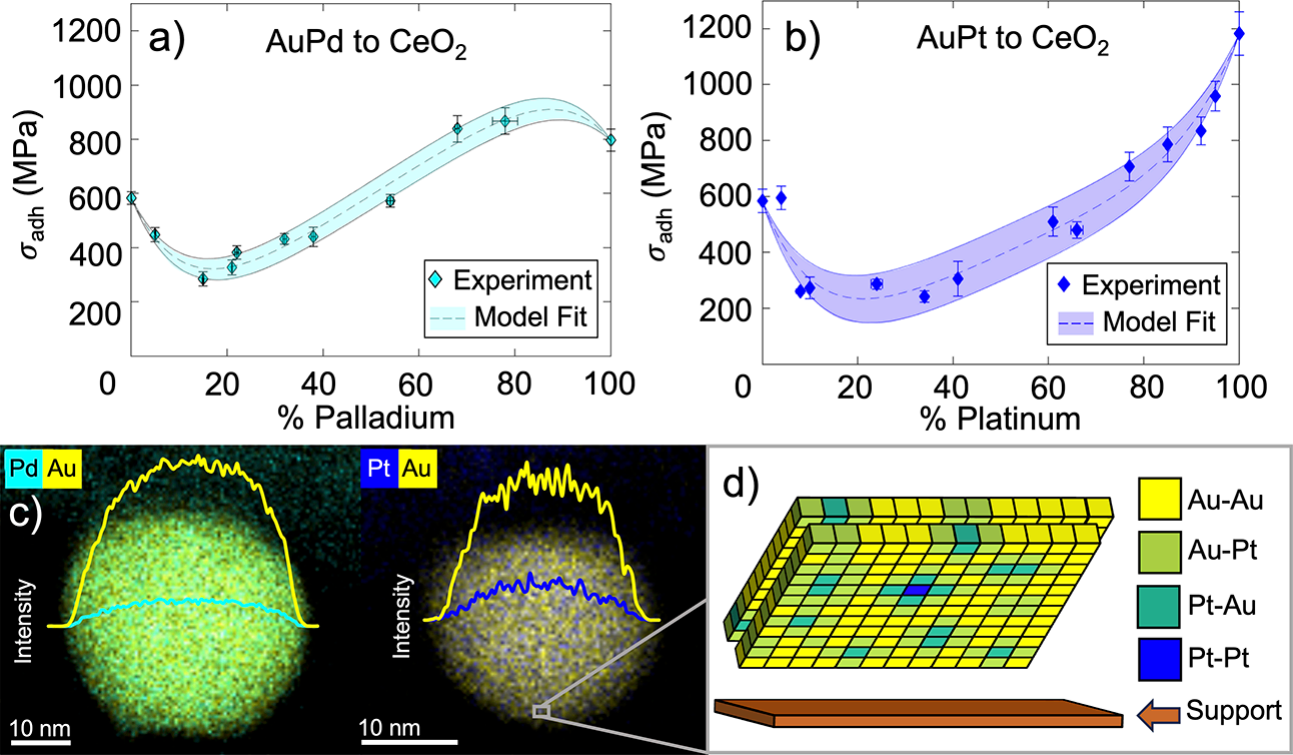
**Adhesion of bimetallic nanoparticles exhibits non-monotonic variation with composition due to charge transfer**. *In situ* measurements of adhesion strength were performed on (a) AuPd and (b) AuPt bimetallic nanoparticles on CeO_2_ across the full range of compositions determined via (c) EDS characterization of the bimetallic nanoparticles. As illustrative examples, nanoparticles with 95 atom % Au and 5 atom % Pd (3c, left) and 92 atom % Au and 8 atom % Pt (3c, right) are shown. (d) The observed non-monotonic dependence of adhesion on composition is then explained with an interface model which considers charge transfer between neighboring atoms in the bimetallic nanoparticle (see main text). The adhesion to the oxide support is modified by electronic interactions of “unlike” nearest neighbors in the nanoparticle (in the schematic, Au next to Pt is indicated by light green, while Pt next to Au is indicated by teal). The best fit of the model is shown (dashed lines in (a,b)), along with bands indicating ± 10% of root-mean-square error (see main text for details).

To explain the non-monotonic trends in adhesion, we aimed for the simplest model that could reproduce the observed experimental behavior and thus yield first insights into properties that govern adhesion of bimetallic nanoparticles (Figure 3d). For this model, stochastic arrays were generated to represent the random distribution of the two metals in the bimetallic nanoparticle surfaces in contact with the supporting oxide material (model compositions were chosen from 0% to 100% Au, in steps of 1%). The overall adhesion strength was determined by summing the adhesion for each surface atom in the array. The subsurface atoms were also considered explicitly, as shown in Figure 3d, because the second-layer atoms also influence the electronic (bonding) environment of their outer-surface neighbors. The (monometallic) adhesion strength of Pt, Pd, and Au in contact with ceria was known from our pure-metal measurements (Figure 2). As expected, a simple rule-of-mixtures approach—i.e. a linear combination of the properties of the two metals—cannot explain the strongly non-monotonic trends. Therefore, charge-transfer effects between dissimilar metals were integrated into the model as it is known that charge transfer between neighboring metal atoms can modify the properties of multimetallic nanoparticles.^29−31^ The impact of intraparticle charge transfer on strength of adhesion was captured by allowing for different adhesion strength of an atom surrounded by at least one dissimilar atom (designated, e.g., as Au–Pt in Figure 3) versus the pure metal, i.e. the same atom surrounded by “like” neighbors (designated as Au–Au). The adhesion strength of an atom with like neighbors was maintained at the value measured in the pure-metal experiments, while the adhesion strength of an atom with unlike neighbors was treated as a fit parameter. Hence, the model has exactly two fit parameters for each bimetallic system, i.e. the unknown adhesion of each metal when surrounded by at least one dissimilar neighbor. These adhesion values were quantified via fitting of the predicted adhesion of the interface model to our experimental data. For full details of the interface model and fitting procedure, see Supplementary Note 3. This simple model resulted in a strong fit with the experimental data (dashed lines in Figure 3a,b indicate the best fit, along with a band of uncertainty that shows all model fittings with root-mean-square error within ± 10% of the error of the best fit). The deterministic fitting routine was further verified using a separate cross-validation approach, described in Supplementary Note 3. The accuracy and uniqueness of the fit, along with the agreement between the two fitting approaches, support the robustness of the fit between the interface model and the experimental measurements.

It can be expected that the interfacial interaction of a metal atom with the support is modified by electron donation from a dissimilar neighboring metal atom, with the direction of the electron transfer determining whether this results in a strengthening or weakening of the interaction. A loss of electron density from one metal should increase its capacity to accept electrons from the support; this could strengthen or weaken its bond, depending on the nature of the metal–support bonding. Accordingly, an increase in electron density will have the opposite effect. Specifically for the present system, it is known that partially reduced ceria transfers electron density to the supported metal nanoparticles,^32−34^ and we confirmed via X-ray photoelectron spectroscopy (XPS) that the present oxide was indeed partially reduced (see Supplementary Note 4). Therefore, an *increase* in electron density in a metal atom should *reduce* charge transfer from the oxide, thus weakening the adhesion between that atom and the supporting oxide; by contrast, a *decrease* in electron density in the neighboring (electron-donating) atom would *increase* its adhesion to the support. We find that this is indeed the case for AuPd: the model fit to the experimental data yields an increase in adhesion strength for Pd from the pure-metal value of 797 ± 41 MPa to a value of 1164 ± 60 MPa for Pd–Au atoms. At the same time, the adhesion strength of Au weakens from 583 ± 22 MPa in Au–Au to 22 ± 43 MPa for Au–Pd (see Table S1 for more details). This is in agreement with prior reports showing electron donation from Pd to Au in the AuPd system.^35,36^ We further confirmed this direction of electron transfer in the AuPd system using a combination of XPS and CO-adsorption experiments (see Supplementary Note 5). This behavior hence explains the S-shaped dependence of the adhesion strength on composition in the AuPd system (Figure 3a).

The composition-dependent adhesion strength of the AuPt system, by contrast, shows a simple concave behavior. Fitting the interface model yields weakened adsorption strengths of *both* Au and Pt in the alloy, with Pt weakening from 1183 ± 78 MPa for the pure metal (Pt–Pt) to 780 ± 93 MPa for Pt–Au, and with Au weakening from 583 ± 22 MPa in Au–Au to near-zero adhesion (0 ± 97 MPa) for Au–Pt in the bimetallic (see Table S1 for more details). This result is surprising in the context of the simple electron-exchange picture that applied to AuPd; however, it agrees with prior reports which show that, due to hybridization of outer electron orbitals, both Au and Pt exhibit a gain in electron density of their outer orbitals in their respective binary alloy.^37−40^ The results are again further supported by a combination of XPS and CO-adsorption measurements which confirm the electronic modifications of the two metals in these binary alloys (see Supplementary Note 5).

Overall, these measurements give a first glimpse into the richness of the physics of multimetallic nanoparticles by revealing a complex, non-monotonic dependence of adhesion of supported bimetallic nanoparticles on composition, governed by charge transfer between dissimilar atoms. The resulting changes in electron density of interfacial metal atoms alter the bonding to the support, which will strengthen or weaken adhesion depending on the direction of electron transfer in the specific metal–support system. The ability to directly measure and quantify these effects will guide the way toward predicting, and ultimately tuning, adhesion of bimetallic nanoparticles. More generally, the flexibility of this new technique—i.e. the *in situ* measurement of nanoparticle-support interactions on an individual nanoparticle level—will enable advances in the understanding of the physics of supported multimetallic nanoparticle systems by exploring the dependence of adhesion on orientation, shape, and morphology of the nanoparticle; by enabling the study of amorphous, polycrystalline, or multioxide supports; and by opening up the vast materials space of multimetallic nanoparticles.

## Materials and Methods

### Nanoparticle Synthesis

Metal nanoparticles were synthesized via wet impregnation directly onto TEM-transparent wedge-shaped silicon substrates. First, a 5-nm layer of ceria was deposited using RF sputter deposition (NexDep Series, Angstrom Engineering, Cambridge, ON) onto silicon wedge samples designed for TEM use (<200 nm Plateau Wedges, Bruker, Billerica, MA). Then, metal nanoparticles were synthesized using a 50 μL solution containing a gold precursor ([Au(en)_2_]Cl_3_), a platinum precursor (Tetraammineplatinum(II) nitrate), or a palladium precursor (Tetraamminepalladium(II) chloride) for synthesizing Au, Pt or Pd nanoparticles, respectively. Concentrations of all solutions were ∼0.001 mol/L for synthesizing monometallic nanoparticles. After drying in a vacuum oven, the wedge was calcined in 0.05 standard cubic centimeter (SCCM) of air flow (*p*_O_2__ = 0.2 atm) at 500 °C for 1–4 h and then reduced in a H_2_ environment (0.5 SCCM of 10% H_2_ in Ar flow, *p*_H_2__ = 0.1 atm) at 180–250 °C for 1–2 h. To synthesize bimetallic nanoparticles, a similar approach was used, using coimpregnation. First, precursors of the individual metals were dissolved at the targeted molar ratio in DI water to achieve a ∼ 0.001 mol/L solution. Then, 50 μL of the solution was placed on the wedge and the wedge was dried in a vacuum oven for 12 h. The wedge was then calcined at 500 °C for 1–4 h (in 0.05 SCCM of air, *p*_O_2__ = 0.2 atm) and reduced in a H_2_ environment at 180–250 °C (0.5 SCCM of 10% H_2_ in Ar flow, *p*_H_2__ = 0.1 atm) for 2 h. After synthesis, the resulting nanoparticles were characterized using TEM before use in the *in situ* adhesion testing. By testing a wide array of times, temperatures, and compositions, we were able to achieve the desired range of particle size (15–40 nm) and compositions (0–100% Au). While wet impregnation is well-known to yield relatively poor control over particle size and composition, the resulting wide distribution of nanoparticles enabled measurement of adhesion strength for multiple sizes and compositions on a single synthesized sample, emphasizing the unique ability of the *in situ* technique to select and probe individual, well-characterized nanoparticles with a desired size and composition.

### *In Situ* TEM Adhesion Tests

Nanoparticle-oxide adhesion tests were performed inside of a transmission electron microscope (Titan Themis G2 200, ThermoFisher Scientific, Waltham, MA) at an accelerating voltage of 200 kV. To avoid effects from the electron beam, the electron dosage was maintained below 7,000 e/Å^2^s (corresponding to 11 A/cm^2^); exposure at this level was demonstrated, in other contexts,^41^ to have identical behavior to when the beam was turned off entirely. Moreover, in the present testing, the precise value of the beam current varied between tests and we saw no detectable effect on results. The TEM was maintained at a vacuum level of <10^–7^ Torr. Nanoparticle samples, created as described in the previous section, were positioned on the stationary sample platform of an *in situ* nanomanipulator (Biasing Manipulator model 1800, Hummingbird Scientific, Lacey, WA). Additionally, 5-nm-thick polycrystalline oxides layers (verified via TEM) were deposited via sputter deposition (NexDep Sputter Deposition System, Angstrom Engineering, Cambridge, ON) on AFM probes (PPP CONT-R, NanoAndMore, Watsonville, CA), which served as cantilever load sensors. The AFM probes had spring constants of approximately 0.5 N/m, but the precise spring constant was calibrated for each individual probe using the Sader method.^42^ The sharp, oxide-coated substrate was brought into contact with the nanoparticle. By piezo-controlled motion, the oxide-coated AFM tip retracts approximately 1 nm/s away from the nanoparticle until the interface is separated resulting in displacement of the tip. Note that this test apparatus enables direct visualization of the surfaces in contact; the present testing did not show plastic deformation of the particles during the separation process. Using frames from immediately before and immediately after separation, the adhesion force was computed from the distance traveled by the cantilever during separation,^20^ as shown in Figure S1.

### Composition Analysis

Immediately after adhesion testing, the composition of the nanoparticles was characterized using a Windowless Super-X EDS detector system (ThermoFisher Scientific, Waltham, MA). Specifically, for the main text, the composition was characterized using a region of interest (ROI) comprising a 5-nm-thick arc that encompasses the contact area. In order to verify that the results were not overly affected by spatial variations in composition, we also tested other shapes, including a full semicircle describing the outermost half of the particle, and also an extremely thin arc, with a thickness of just 1 nm, as shown in Figure S3a. The results of composition-dependent adhesive strength changed very little depending on the ROI used, as shown in Figure S3b,c. In fact, the average change in composition between the shapes was just 4.1 ± 1.4 at% when a 1-nm arc was used, and just 5.4 ± 0.8 at% when a semicircle was used. When we rerun the full analytical analysis using the compositions measured from alternate ROIs, the best-fit parameters change slightly, as shown in Table S1, but all changes are within the uncertainty of the measurement. Based on these analyses, we conclude that the chosen nanoparticles constitute a random mixing with homogeneous composition, and thus the results are not significantly affected by the chosen location and shape of ROI for composition analysis.

## Data Availability

The data that supports this publication has been deposited in the University of Pittsburgh’s institutional repository and is accessible via DOI: http://dx.doi.org/10.18117/m1xf-9p55.

## Supporting Information Available

Additional experimental details, comparison of adhesion values to literature, description of adhesion model, and follow-up characterization of charge transfer (PDF)
A representative *in situ* TEM video of a nanoparticle-oxide adhesion test: Au NP to CeO_2_-coated tip, 5× speed (MOV)

## Supplementary Material

nl5c00076_si_001.pdf

nl5c00076_si_002.mov
