# Supplementary material for: In Situ Measurement of Adhesion for Multimetallic Nanoparticles
Source: Nano Lett. 2025 Apr 15;25(17):6903–9. doi: 10.1021/acs.nanolett.5c00076 (PMC12046597; doi:10.1021/acs.nanolett.5c00076)
Supplement: Supplementary file 1 — nl5c00076_si_001.pdf [file nl5c00076_si_001.pdf]

Supporting Information for the article titled:

In situ measurement of adhesion for multimetallic nanoparticles

Authors

Andrew Baker[1,3], Sai Bharadwaj Vishnubhotla[1], Sanjana Karpe[2], Yahui Yang[2], Götz Veser[2], and Tevis D. B. Jacobs[1]*

Affiliations

[1] Department of Mechanical Engineering and Materials Science, University of Pittsburgh; Pittsburgh, Pennsylvania, 15260, United States of America

[2] Department of Chemical and Petroleum Engineering, University of Pittsburgh; Pittsburgh, Pennsylvania, 15260, United States of America

[3] Center for Integrated Nanotechnologies, Sandia National Laboratories; Albuquerque, New Mexico, 87123, United States of America

* Corresponding authors: Tevis Jacobs: tjacobs@pitt.edu; Götz Veser: gveser@pitt.edu

This PDF file includes:

- Caption for Supplementary Movie S1
- Supplementary Text
- Figs. S1 to S9
- Table S1

Other Supplementary Materials for this manuscript include the following:

- Supplementary Movie S1

Caption for Supplementary Movie S1: The Supplementary Movie S1 shows the approximately 5X-speed video that is captured during the *in situ* transmission electron microscopy-based adhesion tests, as well as a graph of the instantaneous contact force as a function of time.

Supplementary Text:

Supplementary Note 1: Adhesion tests and their relationship to intrinsic interfacial properties

As described in the main text, the primary method established in this investigation is an *in situ* TEM adhesion test. A representative test is shown in the Supplementary Movie S1. Screen shots from this video, as well as annotations indicating the time-stamps and the relevant measured and calculated quantities are shown in Figure S1. Adhesion experiments are well established in mechanics and materials science, including with whole textbooks describing the mechanics of interactions of surfaces in contact.[1] One of the present authors has written extensively on the topic of how to apply these concepts at the nanoscale.[2-6]

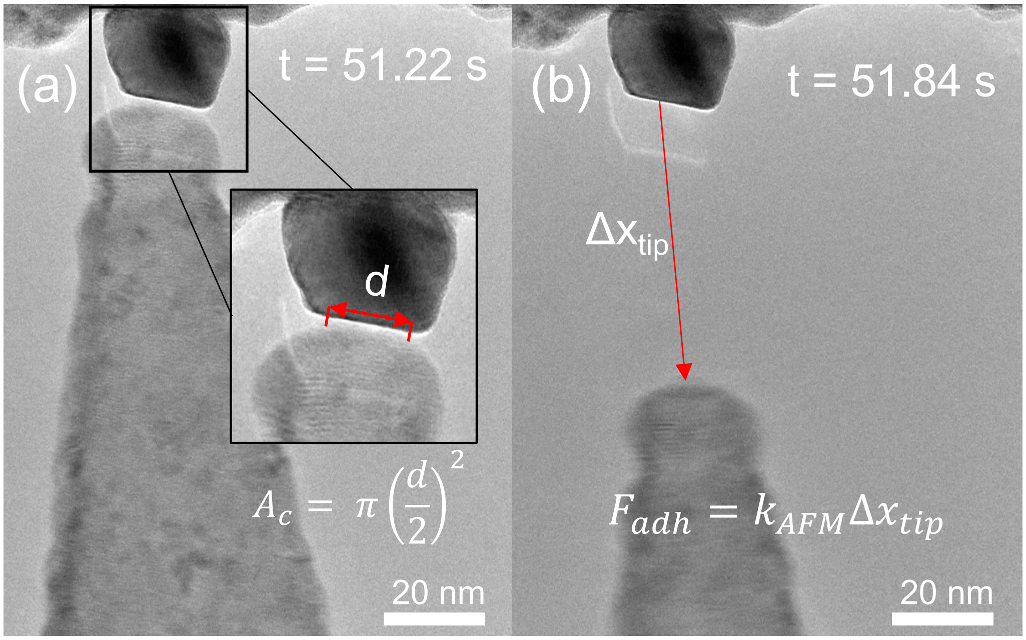

Figure S1. The *in situ* transmission electron microscopy-based technique enables measurement of contact size and adhesion force. (a) During a typical *in situ* TEM nanoparticle adhesion test, the contact diameter is measured right before the moment of separation of the nanoparticle and oxide-coated AFM tip, and then used to estimate a circular area of contact. **(b)** Immediately after separation, the displacement of the oxide-coated AFM tip is measured and used, with the previously measured value of cantilever stiffness, to determine the force required to separate the interface of the nanoparticle and oxide layer.

From a fundamental thermodynamic perspective, the interactions between two surfaces can be described by two directly proportional intrinsic properties, the energy of adhesion and the strength of adhesion.[7] Specifically, the force between two atoms or surfaces is equal to the negative spatial derivative of the energy between these atoms or surfaces at a given separation distance. The adhesion energy (with units of [J/bond] for individual atoms bonded together, and [J/m^2] for surfaces in contact) is the total energy required to separate those atoms (or surfaces) from their equilibrium separation to a fully separated state. The adhesion strength (with units of [N/bond] for individual atoms bonded together, and [N/m^2] for surfaces in contact) represents the maximum force required during this separation.

Importantly, in order to relate the "pull-off force" that is measured in an experiment to intrinsic properties of the interface, it is necessary to understand the mechanism of separation. Adhesive separation is most commonly described using fracture-mechanics concepts. Specifically, the classical contact-mechanics models (especially those of JKR[8] and Maugis[9]) assume crack-like separation at the interface. In these, the intrinsic property of interest is the adhesion energy, also commonly referred to as the "work of adhesion", with units of (J/m^2). However, an additional, separate regime of separation exists which is described by "pop-apart" behavior. Extensive theoretical and experimental investigations in the mechanics community[10-18] have demonstrated the existence of a non-crack-like (also called "flaw-insensitive") limit of behavior, where the interfaces do not separate in a crack-like manner, but instead separate uniformly by a singular "pop-off" event (which is then insensitive to the presence of interfacial flaws, and can no longer be described using the classical models mentioned above). In this regime the force per unit area of separation corresponds to the strength of adhesion (N/m^2).

Recent work by some of the present authors[6] applied the theoretical framework developed by Mesarovic and Johnson[10] to determine which regime applies to nanoscale adhesion experiments. This work computed the governing regime for a wide variety of tests on two different material systems and demonstrated that the majority of tests lay in the pop-off regime. While the full Mesarovic and Johnson model could be applied to the present tests, a simple way to demonstrate that pop-off behavior is governing the present experiments is to show the proportionality between the measured values of contact area and force of adhesion - as shown in Figure 2a,b of the main text.

As mentioned above, these two properties *of the interfaces*, adhesion energy and adhesion strength are proportional, where the adhesion energy is the integral of force over distance and the adhesion force is the maximum of the spatial derivative of energy.[7] Under the simplest possible assumption of a square-well (or "Dugdale") potential, the adhesion energy and the adhesion strength are related by $E_{\mathrm{adh}} = d\sigma_{\mathrm{adh}}$, where d is the distance of interaction. Under more realistic assumptions, the interatomic potential between atoms will not be described by a square well but will instead be governed by more complex electronic interactions. While the proportionality between these two quantities will be maintained, the meaning of the proportionality constant—an "effective distance"—is less clear in this case (this is further discussed in Supplementary Note 2).

Supplementary Note 2: Comparing Adhesion Results for Monometallic Nanoparticles

The trends in nanoparticle adhesion for monometallic systems reported in Figure 2 are used to validate the novel technique against previously reported state-of-the-art measurements.[19,20] These comparisons were conducted as follows.

First, the size-dependence of nanoparticle-support interactions was considered. As mentioned briefly in the main text, prior experimental measurements have shown that the strength of nanoparticle-support interactions becomes size-dependent below approximately 6-nm particle size.[21,22] To avoid this size-dependence, we hence restricted our measurements to larger nanoparticles (15-40 nm). Thus, when comparing our measured adhesion against literature values,

we used values for the largest-size particles that have been previously reported. In addition to the influence of nanoparticle size on adhesion, any mechanical deformation of the nanoparticles would also have been directly observed. In separate work by some of the present authors, we have investigated the mechanical deformation of nanoparticles (for example, Refs.[23,24]) Future investigations hold the potential to relate nanoparticle deformation to nanoparticle adhesion properties. However, the present investigation was confined to cases where mechanical deformation of the nanoparticle was negligible.

Second, while we conducted systematic sets of experiments for selected oxides in contact with a single metal and for varying metals on a single oxide, not all of these systems have been measured previously. For the set of experiments with a constant metal on select oxides (Au on CeO_2, TiO_2, MgO, see Figure 2a), the same systems have been measured previously,[19,25-28] enabling direct comparison. However, for the set of experiments with varying metals on a fixed oxide (Au, Ag, Pt, Pd on CeO_2; Figure 2b), only Au-CeO_2 and Ag-CeO_2 have been reported before; comparison data for Pd-CeO_2 and Pt-CeO_2 are unavailable in the published literature. Nevertheless, we chose the CeO_2 support as the main focus of this set of experiments for three reasons: technologically, it is widely used in catalytic applications;[29,30] scientifically, it is of fundamental interest due to its strong metal-support interactions (SMSI);[28,31-33] and practically, it has higher values of adhesion, resulting in a higher signal-to-noise ratio and more accurate inputs to the analytical model. Therefore, to make the comparisons needed in Figure 2c, we present the adhesion measurements against previously reported values of the same metals (Pt, Pd, Au, and Ag) on a different substrate: MgO. This is reasonable because the metal-to-oxide adhesion depends strongly on the metal's oxophilicity, and it has been shown that a set of metals will show the same trends in behavior across various oxides.[19] Specifically, the ordering of strength from weakest to strongest is expected to be preserved across oxides, and the relative changes in strength should also be proportional. For these reasons, the data in Figure 2c is expected to be well-fit with a line having a non-zero y-intercept. This expected trend is borne out in Figure 2c of the main text.

Third, because our novel technique enables the first-ever direct *in situ* measurement of adhesion force and contact area for nanoparticles, we are able to directly compute the adhesion *strength*, whereas prior reports instead compute adhesion *energy*. As discussed in Supplementary Note 1, these two properties typically scale together by parameter d_{eff};[9,11,34] for which we obtain an average value of 3.8 nm. While this value is clearly much larger than the length over which bonds stretch, the obtained trends in adhesion match literature trends, indicating that the adhesion strength of each system scales linearly with E_{adh}. To avoid adding additional assumptions and uncertainty to the *in situ* TEM method, we directly compare the present values of adhesion strength against previously reported values for adhesion energy. In both cases of Figure 2c, the best-fit lines have R^2 values above 0.9, with $R^2 = 0.97$ for a single metal on various oxides; and $R^2 = 0.92$ for various metals on a single oxide, demonstrating a correlation between previously reported adhesion energy values and the presently reported adhesion strength values. A key advantage of the adhesion strength parameter is that no assumptions about material properties are required when determining the value.

With the data presented in Figure 2 of the manuscript, we hence validate the ability of the *in situ* measurement of nanoparticle-support interactions to accurately measure adhesion between metal nanoparticles and their oxide supports by recreating expected trends from reported values in prior literature.

Supplementary Note 3: Creation, Implementation, and Cross-Validation of the Interface Model

S3.1 Creation and implementation of the Interface Model

To understand the nonmonotonic behavior in bimetallic nanoparticle adhesion, an interface model was created. The aim of this modeling was to identify the simplest model that could explain the observed behavior with a minimum number of assumptions and mathematical fit parameters. The key assumption of the model is that the charge transfer between dissimilar metal atoms in a bimetallic nanoparticle modifies electronic structure and thus adhesion bonding. Therefore, while a gold atom that is fully surrounded by gold atoms will have the same adhesive interaction to CeO_2 that was measured for the monometallic system (Figure 2), a gold atom that has at least one dissimilar neighboring atom (Pt or Pd) will have a modified adhesive interaction with the oxide. Based on this model, two-dimensional arrays (Figure S2) were created to describe the surface and subsurface layers of the mono- and bimetallic nanoparticles at the interface with the support material, and these surface arrays were used to interpret how intra-particle charge transfer between neighboring atoms could modify adhesion and result in the observed nonmonotonic behavior.

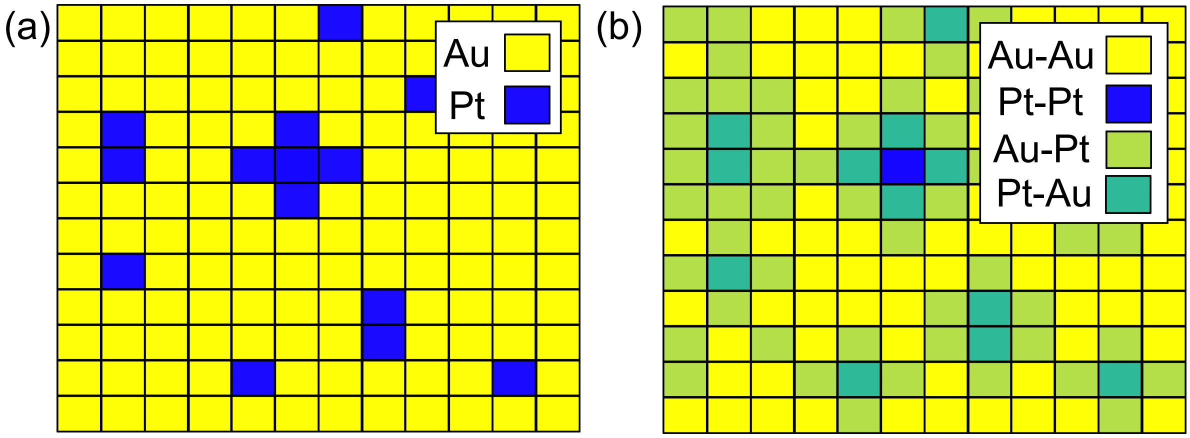

Figure S2. An analytical model, describing charge transfer between nearest neighbors in stochastic arrays of metal atoms, has been applied to explain the experimental results. (a) An example surface array is shown with a stochastic arrangement of Pt (blue squares) and Au (yellow squares) for a bimetallic nanoparticle surface containing 90% Au and 10% Pt. **(b)** Electronic charge transfer is taken into account by coding each of these atoms according to its nearest neighbors: a gold atom completely surrounded by gold (designated Au-Au) is shown in yellow, while a gold atom with at least one Pt neighbor is shown in light green (designated Au-Pt). Likewise, a Pt atom completely surrounded by Pt is colored blue, while at least one gold neighbor would make the atom turquoise. Each of the four different "types" of atoms can take on a different strength of adhesion with the substrate, where the values for the pure "Au-Au" and "Pt-Pt" atoms are known from direct experimental measurement (as shown in Figure 2). (For clarity, only a small (12x12-atom) portion of a surface array is shown; for the analytical calculations, larger arrays are used to reduce the variability of results due to random fluctuations in the patterns, as discussed below.)

First, each position in a two-dimensional array represents an atom in the surface or subsurface layer. As an illustrative example, one such array is shown graphically in Figure S2a, where the positions with a value of zero are shown in yellow color, representing gold, and the positions with a value of one are shown in blue, representing platinum. This process was repeated for the second layer of atoms (not shown in Figure S2.) All arrays were generated with a stochastic distribution of ones and zeros, where the total area fraction corresponds to a given bulk composition of the nanoparticle. This process was repeated for the full range of compositions for the bimetallic nanoparticle from 0% to 100% Au in steps of 1%. The specific example of Figure S2a shows 90% Au and 10% Pt, where the surface layer (shown) and the sub-surface layer (not shown) each have 90% of the positions having values of 0 and the other 10% having values of 1.

Second, to integrate the key assumption that intra-particle charge transfer modifies adhesion, two additional categories of atoms were created to represent the atoms with modified adhesion strength. These additional categories represent Au in contact with a Pt neighbor (designated Au-Pt) and Pt in contact with an Au neighbor (designated Pt-Au). In Figure S2b, these atoms are represented using light green and turquoise color, respectively. If at least one of the 8 nearest neighbors for an FCC surface atom (four neighbors in the surface layer and four neighbors in the sub-surface layer) is of a dissimilar metal, then this atom is designated as having modified adhesion (i.e., Au-Pt or Pt-Au). The same process is applied to the AuPd system, and the resulting surface arrays are then used to account for charge transfer between metals as described quantitatively below.

Third, as described in the main text, measurements were performed of the composition of each tested bimetallic nanoparticle; these served as inputs to the model, giving the relevant composition to analyze for that particle. Because the model assumes random mixing, we attempted to verify the accuracy of this assumption by varying the size and position of the region of interest (ROI) used for compositional analysis. The results are shown in Figure S3 and Table S1, and demonstrate that there was no significant change in measured composition with the ROI used. This serves to demonstrate that the results of this investigation are robust to the method, and do not depend strongly on where the compositional analysis is performed. Furthermore, this validates the assumption of the interface model that there is random mixing of the alloys. Prior investigations have shown that these alloys are known to form either core/shell structures or randomly mixed alloys, depending on synthesis and annealing conditions.[35,36] The present investigation was focused on understanding charge-transfer effects on adhesion, and therefore randomly mixed alloys were preferrable.

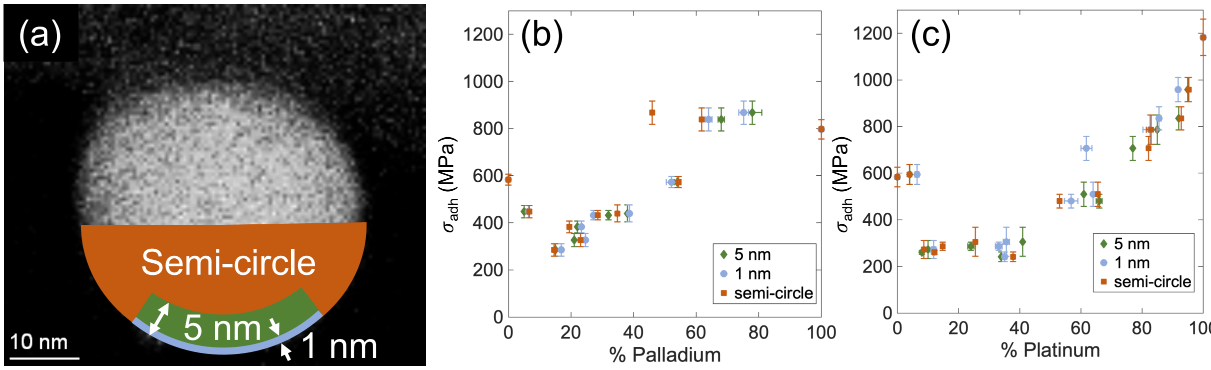

Figure S3. Energy dispersive x-ray spectroscopy (EDS) was used to determine the composition of the bimetallic particles. (a) To determine the surface composition of the nanoparticles from EDS images, different areas were selected from which the EDS signal was collected: a semi-circle, a 5-nm-thick arc, and a 1-nm-thick arc. Adhesion strength was plotted against the resulting compositions for each ROI for (b) the AuPd system and (c) the AuPt system. The results demonstrate that the trends do not vary significantly with the region of interest chosen for analysis, suggesting that surface atoms are randomly distributed. For Figure 3 in the main text, the 5-nm fit was chosen, in order to balance EDS signal strength and spatial resolution.

Table S1. Minor variation in best-fit parameters with variation in the region-of-interest for analysis (values with units of MPa).

	5 nm fit	Error	1 nm fit	Error	Semicircle fit	Error
**Au-Pt**	0	97	0	115.5	42	92
**Pt-Au**	780	93	889	116.6	758	96
	**5 nm fit**	**Error**	**1 nm fit**	**Error**	**Semicircle fit**	**Error**
**Au-Pd**	22	43	16	59.5	9	145
**Pd-Au**	1164	60	1205	80	1353	215.5

Following this framework, arrays were created for the entire range of composition for both AuPt and AuPd nanoparticles. The relative amounts of each type of atom (i.e. Au-Au, Au-Pt, Pt-Au, or Pt-Pt) were computed for each composition, and are shown in Figure S4. For pure or near-pure compositions, almost all of the atoms are surrounded by like atoms (Au-Au or Pt-Pt), while compositions near the midpoint contain large fractions of Au-Pt and Pt-Au.

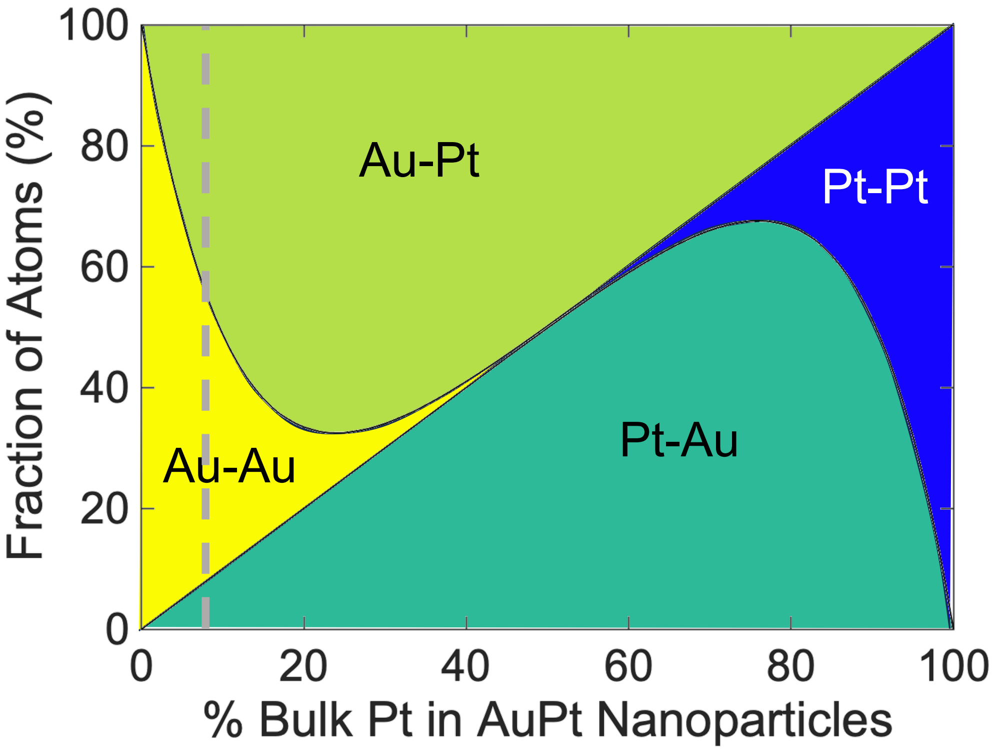

Figure S4: The fraction of atoms that are surrounded by atoms of the same species or by dissimilar neighbors varies with the (bulk) composition of the alloy. The relative amounts of each type of metal atom are computed as described above and plotted against the (bulk) amount of Pt. While the overall amount of each metal (Au or Pt) is fixed by the bulk composition, the proportion of their neighbor types (e.g., Au-Au or Au-Pt) is computed from the stochastic arrays. For example, at a bulk composition of 7% platinum (gray dashed line), all 7% of the Pt atoms sit next to at least one Au atom and so all are designated Pt-Au. However, the gold is split between like atoms and unlike atoms. Approximately 40% of all atoms are gold with at least one platinum neighbor; the remaining 53% (which is equal to 100% - (7%+40%)) of all atoms consists of gold sitting next to only gold atoms.

With charge-transfer effects integrated into the surface/sub-surface arrays using the four types of atoms, the overall adhesion strength of an array surface ($\sigma_{adh-model}$) can be determined from a weighted sum of the individual atomic adhesion strengths:

$$\sigma_{adh-model} = x_{Au-Au}\sigma_{Au-Au} + x_{Au-Pt}\sigma_{Au-Pt} + x_{Pt-Au}\sigma_{Pt-Au} + x_{Pt-Pt}\sigma_{Pt-Pt} \qquad (S1)$$

where x_i and σ_i represent the (fractional) composition and adhesion strength of the ith atom type. As mentioned, the compositions are computed from stochastic arrays (Figure S3), while the pure-metal adhesion strengths (σ_{Au-Au} and σ_{Pt-Pt}) are known from the measurements using monometallic nanoparticles shown in Figure 2. The only two unknowns in Equation S1 are the atomic adhesion strengths of the atoms with dissimilar neighbors (σ_{Au-Pt} and σ_{Pt-Au}). These two values are used as fitting parameters. Fitting was initially conducted with array sizes of 100x100 atoms to approximate the actual size of the bimetallic nanoparticle surfaces in the experiments. Later, to verify that the model is insensitive to array size, very large surface arrays (1500x1500

atoms) were created and analyzed as well. Fit results were compared to those with the smaller 100x100 array and showed less than 1% difference in values for $\sigma_{\mathrm{Au-Pt}}$ and $\sigma_{\mathrm{Pt-Au}}$.

The final step of the model is to fit experimental data with the model by varying values of $\sigma_{\mathrm{Au-Pt}}$ and $\sigma_{\mathrm{Pt-Au}}$. A simple recursive fitting routine was used that explores all possible values of adhesion strength of unlike metals ranging from 0 MPa (representing a total loss of adhesion to the substrate in the case of dissimilar neighbors) up to 1500 MPa (representing increased substrate interactions for metal atoms with dissimilar neighbors). The quality of the fit was assessed by computing the root-mean-square error (RMSE) between the predicted values and those measured by experiment. The parameter space was searched, first using a coarse increment of 1 MPa, and subsequently with finer increments of 0.01 MPa. In all cases, a unique pair of values was found that minimized the RMSE. These best-fit values represent the modified strength of an atom with dissimilar neighbors. In order to assess the uncertainty of these values (and the uniqueness of the fit), we also report the range of results from systems with RMSE up to 10% greater than the best fit. These are shown as an uncertainty band in Figure 3a,b. We see that the values of the fitting parameters do not vary significantly in either system, suggesting that unique values exist for the modified adhesion strength of each type of metal atoms in the AuPt and AuPd systems.

S.2 Cross-Validation of the Interface Model

After performing the deterministic fitting routine described above, an additional cross-validation approach was applied in order to verify the robustness of the obtained results against the choice of fitting routine. Specifically, a rotation estimation based on a leave-one-out approach was implemented in which the input dataset was divided into k folds. Here, k was set equal to the number of data points in each system (AuPt or AuPd, respectively). In each run, a different fold of the data is left out for validation while the remaining folds are used for training the model using nonlinear least-squares fitting. The runs are repeated k times (i.e., the number of folds). In this way, an ensemble of parameters and their associated validation errors are obtained from each run.[37] The final parameter set, $\sigma_{\mathrm{Au-Pt}}$ and $\sigma_{\mathrm{Pt-Au}}$, is then calculated from the fit parameters of all runs, weighted based on their respective validation errors using a quadratic weight function widely used for nonparametric regression:

$$\Theta = \sum_{u=1}^{k} \omega_u \theta_u,\ \omega_u = \frac{1/E_u^2}{\sum_{u=1}^{k} 1/E_u^2}$$

where Θ is the fitted parameter of each run, ω is validation error weight, E is the validation error of each run, and k is the number of folds.[38]

Using this cross-validation approach for the AuPd bimetallic system, an adhesion strength for modified Au of $\sigma_{\mathrm{Au-Pd}}$ = 25 MPa and an adhesion strength for modified Pd of $\sigma_{\mathrm{Pd-Au}}$ = 1161 MPa is obtained. For the AuPt bimetallic system, an adhesion strength of $\sigma_{\mathrm{Au-Pt}}$ = 0 MPa is obtained for modified Au, along with an adhesion strength of $\sigma_{\mathrm{Pt-Au}}$= 760 MPa for modified Pt. All values obtained via cross-validation are within 5% of the deterministic fitting algorithm described in Section S2.1; this result supports the robustness of the fit between the numerical model and the experimentally measured data.

Supplementary Note 4: Oxidation State of the Ceria Support

Depending on its reduction state, ceria can donate or accept electrons from a supported metal nanoparticle.[39] In reduced ceria, oxygen vacancies enhance the electron density of the support, facilitating electron donation to the vacant d orbitals of the metals. Thus, for Au/ceria catalysts, the formation of negatively charged Au sites due to charge transfer from oxygen vacancies to Au has been reported,[40,41] further supported by theoretical investigations which predict the formation of negatively charged gold sites on Au/CeO_{2-x} catalysts.[42] Similarly, a density functional theory (DFT) study of Pd supported on partially reduced CeO_{2-x} confirmed electron transfer from oxygen vacancies to the Pd metal.[43]

We confirmed the presence of partially reduced ceria for our system using x-ray photoelectron spectroscopy (XPS). Figure S5 shows the XPS spectrum of a ceria-coated wedge. The ten peak assignments are labeled according to the convention established by Burroughs, *et al.*[44] The peaks labeled U, U'', U''' and V, V'', V''' represent Ce^{4+} $3d_{3/2}$ and $3d_{5/2}$ states, respectively, while U', V' indicate to Ce^{3+} $3d_{3/2}$ and $3d_{5/2}$, and V^0 and U^0 correspond to the Ce^{3+} $3d^9$ $4f^1$ O $2p^6$ final state. XPS thus confirms the presence of Ce^{3+} alongside Ce^{4+} and hence that ceria is in a partially reduced state. This suggests that transfer of electron density from the ceria support to the supported metal nanoparticles should be expected for the present supported metal nanoparticle systems.

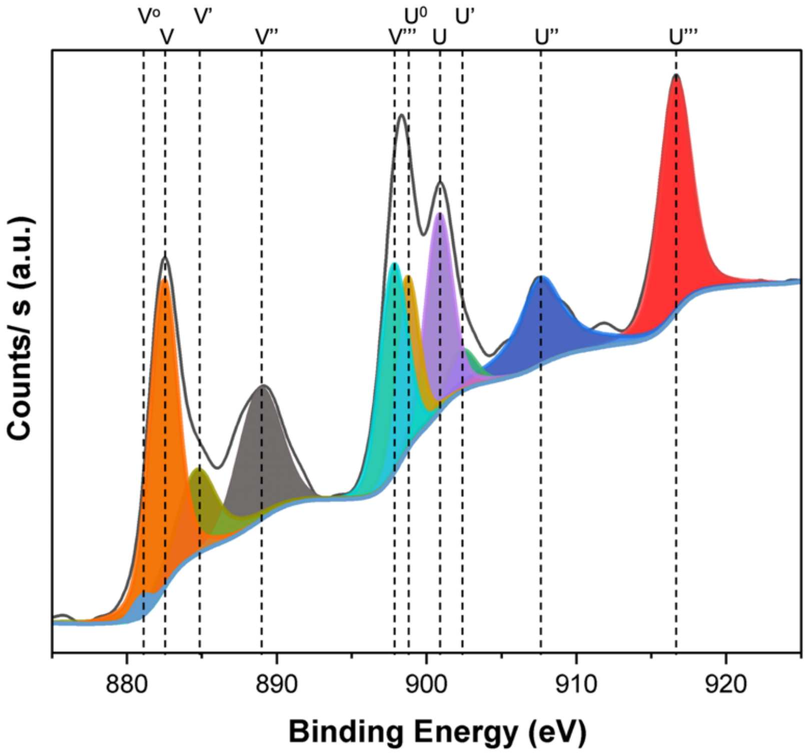

Figure S5: XPS analysis on the ceria-coated wedge confirms that the ceria substrate is in a partially reduced state. Specifically, the presence of Ce^{3+} alongside Ce^{4+} is in agreement with prior literature on ceria. (See text for further discussion on assignment and analysis of the peaks.)

Supplementary Note 5: Experimental Verification of Charge Transfer in the Bimetallic Nanoparticles

Since electron transfer determines the strength of the adhesion between metal nanoparticles and their support,[39,45,46] internal charge transfer between dissimilar metal atoms in a bimetallic nanoparticle is expected to modify adhesion bonding at the interface. Specifically for the present bimetallic systems, an increase in electron density—and hence a decreased ability to accept electrons from the ceria support—should reduce the strength of adhesion for the electron-accepting metal and increase that of the electron-donating metal. In order to verify the direction of charge transfer in the bimetallic nanoparticles between Au and Pd or Pt, respectively, we used a combination of XPS analysis and CO adsorption studies.

S5.1 Experimental Set-Up

CO DRIFTS. To investigate variations in electron density on bimetallic nanoparticles, we used *in situ* diffuse reflectance infrared Fourier-transform spectroscopy (DRIFTS) measurements, using CO as a probe molecule. CO adsorption can serve as a sensitive probe for charge transfer between constituent metals in a bimetallic nanoparticle since the vibrational frequency of adsorbed CO is sensitive to electron donation from the underlying metal and hence its electron density. The experiments employed a liquid nitrogen-cooled MCT detector (Bruker Vertex-70LS FTIR) and an environmentally controlled PIKE DiffuseIR cell with KBr windows at ambient pressure. The measurements were conducted on powdered $AuPt/CeO_2$ and $AuPd/CeO_2$ samples. Spectra were acquired at room temperature, spanning the 400-4000 cm^{-1} range, with a resolution of 4 cm^{-1}. The sample chamber was purged with N_2 flow (20 SCCM), and a background scan was recorded for background subtraction. Subsequently, CO was introduced into the chamber at a flow rate of 20 SCCM, and CO-DRIFTS spectra were collected until no discernible change in the spectrum was observed. Another round of N_2 purge was initiated, and CO-DRIFTS data were recorded until stability in the spectra was achieved.

X-ray Photoelectron Spectroscopy. To assess the reduction state of ceria and determine electron-density changes on bimetallic nanoparticles, XPS was performed on ceria, $AuPt/CeO_2$, and $AuPd/CeO_2$ wedges. The measurements were conducted using a ThermoFisher ESCALAB 250 Xi spectrometer equipped with a monochromatic Al Kα x-ray source at room temperature under vacuum conditions (approximately 10^{-8} - 10^{-9} Torr). The wedges were mounted on copper tape, and an external electron flood gun was used to counteract charge accumulation on non-conductive samples. Spectra were recorded with an x-ray spot size of 650 μm and a step size of 0.1 eV over 43 steps. The C line at 284.8 eV was used as a reference for calibration of binding energy. Curve fitting of XPS spectra was conducted using OriginLab software, employing the Shirley background function for background subtraction.

S5.2 Experimental Corroboration of Charge Transfer in AuPd Bimetallic Nanoparticles

To probe the electron transfer in the AuPd bimetallic system, we used CO-DRIFTS on ceria-supported AuPd nanoparticles. Prior infrared spectroscopy studies of CO adsorbed on supported bimetallic AuPd nanoparticles showed a blue-shift in the bridge-bonded CO to higher wavenumbers with an increase in Au/Pd ratio, indicating charge transfer from Pd to Au [47]. To confirm this direction of charge transfer in our AuPd nanoparticles, we conducted CO-DRIFTS measurements on ceria-supported AuPd nanoparticles with varying compositions; the results are shown in Figure S6 for a range of Au/Pd ratios. The CO-DRIFTS spectrum of monometallic Pd

nanoparticles (shown in red) exhibits two distinctive bands: the band at 1987 cm^{-1} is attributed to linear CO absorption on Pd, while the band at 1925 cm^{-1} is attributed to bridged CO adsorption on Pd atoms [48]. Both linear and bridge-bonded CO show a blue shift of the CO absorption band to higher wavenumbers from 1925 cm^{-1} to 1937.5 cm^{-1} and from 1987 cm^{-1} to 1993 cm^{-1}, respectively, upon alloying with Au (shown in blue for $Pd_{80}Au_{20}$ and in green for $Pd_{20}Au_{80}$ in Figure S6), indicating a loss in electron density for Pd.

In pure Au nanoparticles (shown in black in Figure S6), the absence of CO adsorption bands indicates that CO does not adsorb significantly at room temperature on the large-sized Au nanoparticles, as is well established in prior literature [49].

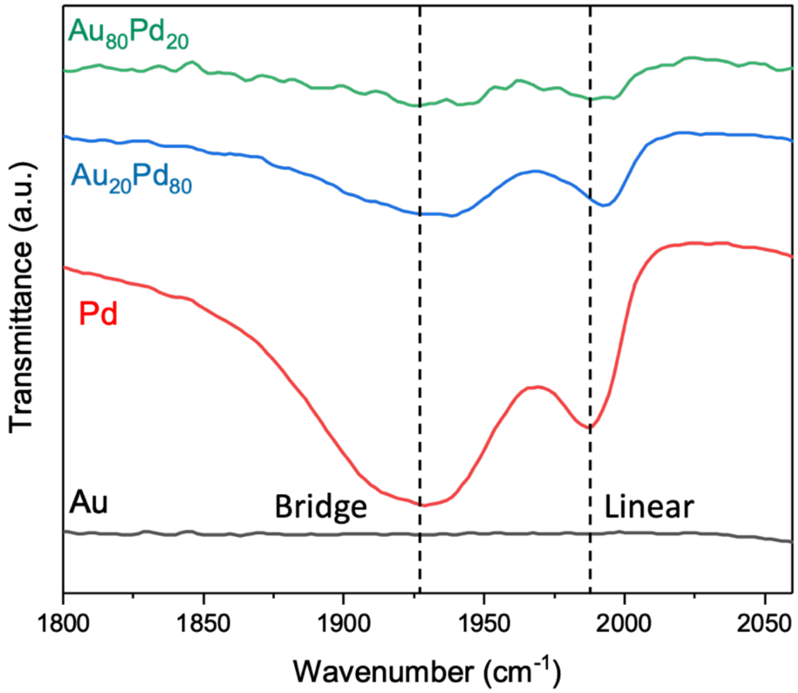

Figure S6: CO-DRIFTS shows changes in the vibration frequency of adsorbed CO with changes in composition of the AuPd particles, indicating charge transfer between the two metals. DRIFTS spectra of CO adsorbed on AuPd/Ceria powder samples with varied bulk composition: monometallic Au and Pd nanoparticles (shown in black and red, respectively), bimetallic $Pd_{80}Au_{20}$ nanoparticles (shown in blue) and bimetallic $Pd_{20}Au_{80}$ nanoparticles (shown in green). The blue shift of the CO IR bands indicates that Pd has a lower electron density in the bimetallic nanoparticles compared to pure Pd particles.

Thus, to confirm the direction of charge transfer for Au in the AuPd bimetallic particles, the CO-DRIFTS analysis was complemented by XPS analysis of ceria-supported AuPd nanoparticles; the results are shown in Figure S7. The XPS spectrum of the ceria-supported monometallic Au sample shows the presence of two peaks, attributed to Au^0 $4f_{7/2}$ (84.0 eV) and Au^0 $4f_{5/2}$ (88.0 eV), respectively,[50] confirming the presence of metallic Au species (Figure S7; shown in orange). These

binding energies shift to lower values upon alloying Au with Pd (see Figure S7: $Pd_{20}Au_{80}$, red; $Pd_{50}Au_{50}$, blue; and $Pd_{80}Au_{20}$, green), indicating an increase in electron density for Au, i.e. electron donation from Pd to Au, and hence a reduced ability of Au to accept electrons from the support.

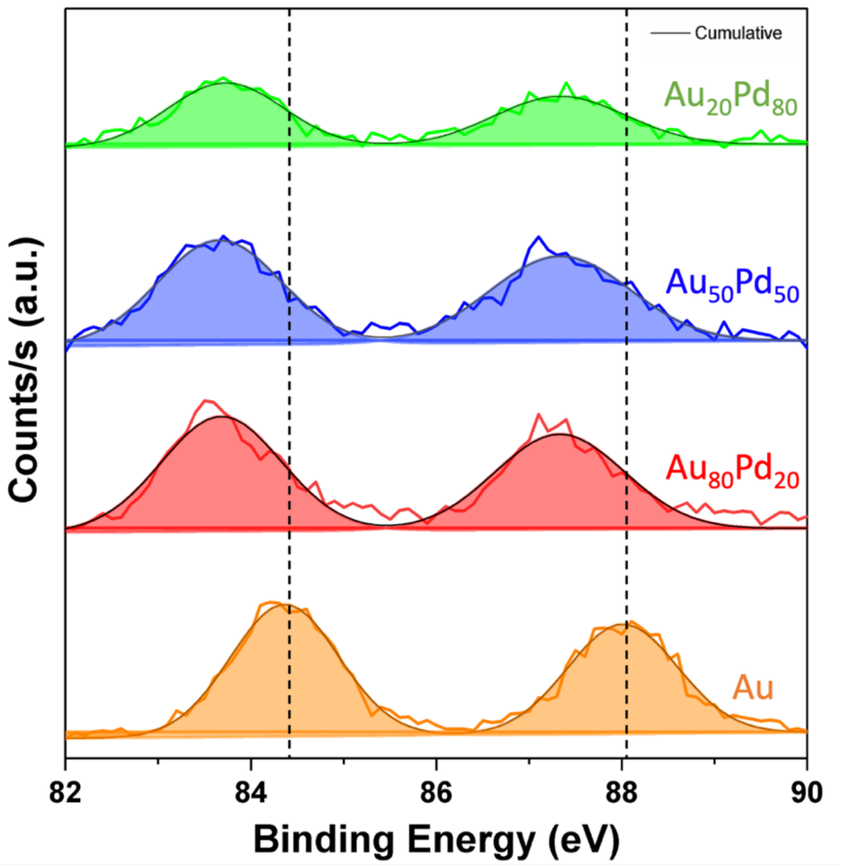

Figure S7: XPS measurement of AuPd nanoparticles confirms electron transfer via corresponding peak shifts. Au 4f region of the XPS spectra of AuPd nanoparticles supported on ceria are shown for various nominal bulk composition (with colors indicated in the image). The negative shift in the Au 4f binding energy upon alloying with Pd (left peak) confirms an increased electron density of Au in the AuPd bimetallic due to electron donation from Pd to Au.

The combination of XPS and CO-DRIFTS analyses hence shows that the direction of electron transfer in these ceria-supported AuPd bimetallic nanoparticles is from Pd to Au, resulting in a decrease in electron density on Pd (confirmed via CO-DRIFTS) and an increase in electron density on Au (confirmed via XPS), in line with expectations from the electronegativity of the metals. Hence as a result of its electron-depletion, Pd is expected to show an increased ability to accept electrons from the partially reduced ceria support, and the strength of adhesion of Pd should increase. Conversely, the electron-enrichment of Au should result in a decreased strength of adhesion for Au. This agrees well with the results from the model fit to our experimental adhesion data, where adhesion of Au decreases from 583.6 MPa for pure Au nanoparticles to 25.27 MPa in the Au atoms surrounded by Pd atoms, while adhesion of Pd increases from 796.8 MPa for pure Pd to 1161 MPa for Pd surrounded by at least one Au atom in the bimetallic nanoparticles.

S5.3 Experimental Corroboration of Charge Transfer in AuPt Bimetallic Nanoparticles

While electron charge transfer in bimetallic nanoparticle systems typically occurs in the direction suggested by electronegativity differences, this behavior has been shown to be more complex in the AuPt system. A simple electronegativity argument would suggest a gain in electron density of Au and a loss in Pt, as also supported for example by a computational study by Mokkath and Schwingenschlögl,[51] and experimental studies by Zeng *et al.*[52] and Bus and Bokhoeven.[53] In contrast, Doherty *et al.*[50] reported an opposite electron transfer from Au to Pt in silica-supported AuPt nanoparticles, supported by evidence from XPS and CO-FTIR. This agrees with Wong *et al.*[54] who reported an increase in electron density (Mullikan charges) for Au and a decrease for Pt in TiO_2-supported bimetallic AuPt nanoparticles based on DFT calculations. However, the experimental XPS data in the same report shows negative shifts in binding energy, i.e. increased electron density on both metals upon alloying, in contradiction not only to the computational results but also in apparent violation of charge neutrality. The resolution to this seeming contradiction comes from recent work which explains similar XPS observations based on a charge-compensation model which accounts for s-p-d hybridization and hence satisfies charge neutrality.[55] Calculation of the projected density of states (PDOS) for Au and Pt atoms in the bimetallic systems (albeit calculated for 55 atom clusters, as opposed to the much larger nanoparticles in these studies as well as ours) indicate a shift of electron density towards the Fermi level and hence a high availability of these electrons with a resulting increase in catalytic activity.[56]

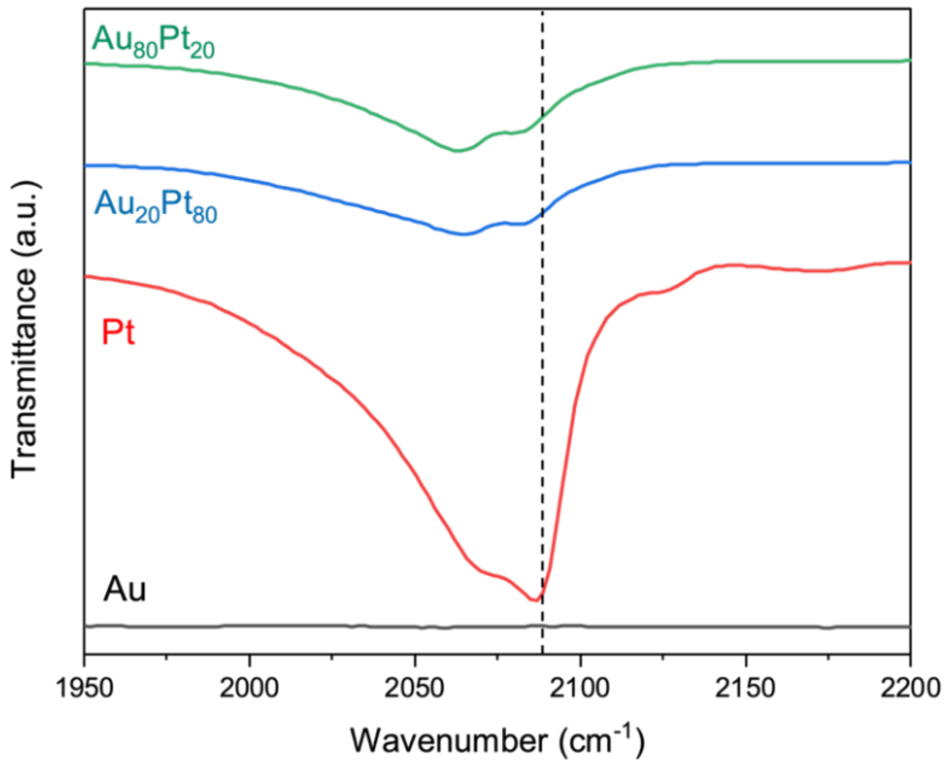

Figure S8: CO-DRIFTS results show a red shift upon alloying Pt with Au. DRIFTS spectra are shown for CO adsorbed on AuPt/ceria powder samples with varied bulk composition: Au nanoparticles (black), Pt nanoparticles (red), $Pt_{80}Au_{20}$ bimetallic nanoparticles (blue), and $Pt_{20}Au_{80}$ bimetallic nanoparticles (green). A red shift (towards lower wavenumbers) indicates an increase in electron density in Pt upon alloying.

In order to validate this increase in electron density on both Au and Pt in AuPt alloy nanoparticles, we again performed CO-DRIFTS and XPS analyses for AuPt nanoparticles on ceria supports. The CO-DRIFTS analysis on AuPt bimetallic nanoparticles with varying Au/Pt ratio is shown in Figure S8. The results confirm a red shift of the CO absorption band from 2075 to 2061 cm^{-1} upon alloying Pt with Au, indicating a gain in electron density for Pt.

Since CO desorbs below room temperature on large Au nanoparticles (as discussed in Section 4.1), the change in electron density of Au upon alloying with Pt in AuPt bimetallic nanoparticles was analyzed via XPS. Figure S9 shows the Au 4f binding energy region of the XPS spectra for AuPt samples with varying Au:Pt composition. Again, two peaks attributed to Au^0 $4f_{7/2}$ (84.0 eV) and Au^0 $4f_{5/2}$ (88.0 eV), are observed for Au/CeO_2, confirming the presence of metallic Au species (see Figure S9; shown in orange). These Au^0 4f peaks shift to lower binding energy in the $AuPt/CeO_2$ bimetallic samples (see Figure S9: $Pt_{20}Au_{80}$, red; $Pt_{50}Au_{50}$, blue; and $Pt_{80}Au_{20}$, green), indicating an increase in electron density for Au in the alloy samples.

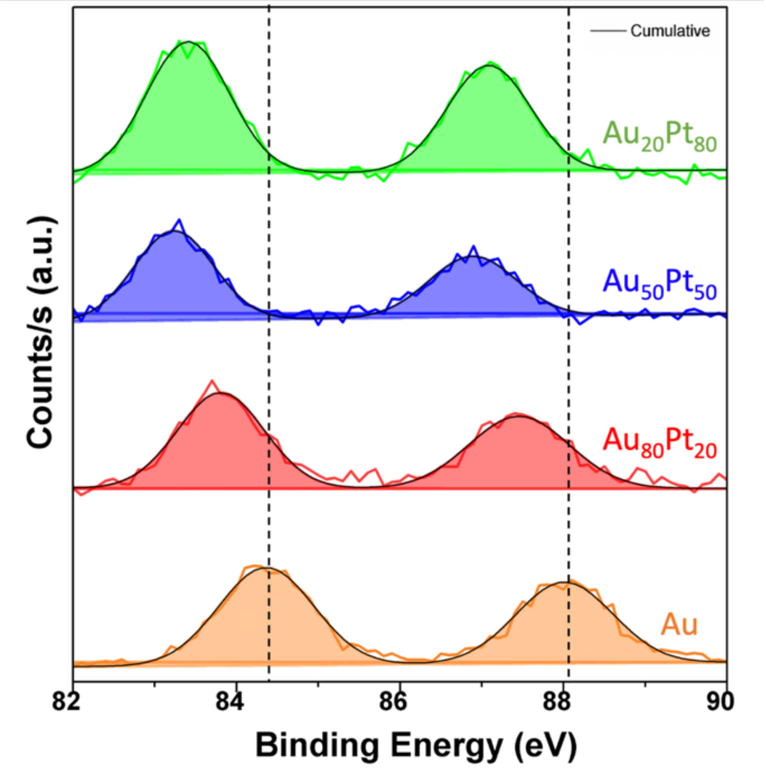

Figure S9: XPS confirms an increase in electron density of Au upon alloying with Pt. The Au 4f region of the XPS spectra is shown for monometallic Au and bimetallic AuPt nanoparticles supported on a ceria-coated wedge with varied bulk composition. The negative shift in the Au 4f binding energy in the bimetallic as compared to the monometallic Au nanoparticles indicates an increased electron density of Au in bimetallic AuPt nanoparticles.

The combined XPS and CO-DRIFTS analyses thus confirm the gain in electron density on both Au (as confirmed via XPS analysis) and Pt (as confirmed via CO-FTIR analysis) in the AuPt bimetallic system, in agreement with the above discussed report by Wu *et al.*[56] Hence, for ceria-

supported AuPt, where adhesion of the nanoparticle occurs via electron transfer from the ceria support to the supported metals, the increased electron density on both Au and Pt is expected to result in weakened adhesion for both metals. This again agrees well with the model fit to our experimental data where adhesion of Au decreases from 583.6 MPa for pure Au nanoparticles to 30.7 MPa of Au next to Pt, and adhesion of Pt decreases similarly from 1182.58 MPa for pure Pt to 759.43 MPa for Pt with Au neighbors.

Overall, our experimental characterization of the bimetallic nanoparticle systems hence supports a rather straightforward and consistent picture that identifies charge transfer between the constituent metals in the bimetallic particle, as well as between particle and support, as the origin of the observed complex trends in strength of adhesion with composition of the bimetallic AuPd and AuPt systems.

References

(1) Johnson, K. L. *Contact mechanics*; Cambridge university press, 1987.
(2) Jacobs, T. D.; Mathew Mate, C.; Turner, K. T.; Carpick, R. W. Understanding the Tip–Sample Contact: An Overview of Contact Mechanics from the Macro-to the Nanoscale. *Scanning Probe Microscopy in Industrial Applications: Nanomechanical characterization* **2013**, 15-48.
(3) Jacobs, T. D.; Carpick, R. W. Nanoscale wear as a stress-assisted chemical reaction. *Nature nanotechnology* **2013**, *8* (2), 108-112.
(4) Jacobs, T. D.; Ryan, K. E.; Keating, P. L.; Grierson, D. S.; Lefever, J. A.; Turner, K. T.; Harrison, J. A.; Carpick, R. W. The effect of atomic-scale roughness on the adhesion of nanoscale asperities: A combined simulation and experimental investigation. *Tribology Letters* **2013**, *50*, 81-93.
(5) Jacobs, T. D.; Martini, A. Measuring and understanding contact area at the nanoscale: a review. *Applied Mechanics Reviews* **2017**, *69* (6), 060802.
(6) Baker, A. J.; Vishnubhotla, S. B.; Chen, R.; Martini, A.; Jacobs, T. D. B. Origin of Pressure-Dependent Adhesion in Nanoscale Contacts. *Nano Letters* **2022**, *22* (14), 5954-5960. DOI: 10.1021/acs.nanolett.2c02016.
(7) Israelachvili, J. N. *Intermolecular and Surface Forces*; Academic Press, 2011. DOI: 10.1016/c2009-0-21560-1.
(8) Johnson, K. L.; Kendall, K.; Roberts, A. Surface energy and the contact of elastic solids. *Proceedings of the royal society of London. A. mathematical and physical sciences* **1971**, *324* (1558), 301-313.
(9) Maugis, D. Adhesion of spheres: The JKR-DMT transition using a dugdale model. *Journal of Colloid and Interface Science* **1992**, *150* (1), 243-269. DOI: 10.1016/0021-9797(92)90285-t.
(10) Mesarovic, S. D.; Johnson, K. L. Adhesive contact of elastic–plastic spheres. *Journal of the Mechanics and Physics of Solids* **2000**, *48* (10), 2009-2033. DOI: 10.1016/s0022-5096(00)00004-1.
(11) Persson, B. N. J. Nanoadhesion. *Wear* **2003**, *254* (9), 832-834. DOI: 10.1016/s0043-1648(03)00233-3.
(12) Hui, C.-Y.; Glassmaker, N.; Tang, T.; Jagota, A. Design of biomimetic fibrillar interfaces: 2. Mechanics of enhanced adhesion. *Journal of the Royal Society Interface* **2004**, *1* (1), 35-48.

(13) Yao, H.; Ciavarella, M.; Gao, H. Adhesion maps of spheres corrected for strength limit. *Journal of colloid and interface science* **2007**, *315* (2), 786-790.
(14) Jiang, Y.; Grierson, D. S.; Turner, K. T. Flat punch adhesion: transition from fracture-based to strength-limited pull-off. *Journal of Physics D: Applied Physics* **2014**, *47* (32), 325301.
(15) Liu, Y.; Gao, Y. Non-uniform breaking of molecular bonds, peripheral morphology and releasable adhesion by elastic anisotropy in bio-adhesive contacts. *Journal of the Royal Society Interface* **2015**, *12* (102), 20141042.
(16) Gao, Z.; Gao, Y. Why do receptor–ligand bonds in cell adhesion cluster into discrete focal-adhesion sites? *Journal of the Mechanics and Physics of Solids* **2016**, *95*, 557-574.
(17) Chen, C.; Wang, Z.; Suo, Z. Flaw sensitivity of highly stretchable materials. *Extreme Mechanics Letters* **2017**, *10*, 50-57.
(18) Long, R.; Hui, C.-Y.; Gong, J. P.; Bouchbinder, E. The fracture of highly deformable soft materials: A tale of two length scales. *Annual Review of Condensed Matter Physics* **2021**, *12* (1), 71-94.
(19) Hemmingson, S. L.; Campbell, C. T. Trends in Adhesion Energies of Metal Nanoparticles on Oxide Surfaces: Understanding Support Effects in Catalysis and Nanotechnology. *ACS Nano* **2017**, *11* (2), 1196-1203. DOI: 10.1021/acsnano.6b07502.
(20) Zhao, K.; Auerbach, D. J.; Campbell, C. T. Predicting Adhesion Energies of Metal Nanoparticles to Support Surfaces, Which Determines Metal Chemical Potential versus Particle Size and Thus Catalyst Performance. *ACS Catalysis* **2024**, *14* (17), 12857-12864. DOI: 10.1021/acscatal.4c02559.
(21) Ahmadi, M.; Behafarid, F.; Cuenya, B. R. Size-dependent adhesion energy of shape-selected Pd and Pt nanoparticles. *Nanoscale* **2016**, *8* (22), 11635-11641. DOI: 10.1039/c6nr02166b.
(22) Campbell, C. T.; Mao, Z. Chemical Potential of Metal Atoms in Supported Nanoparticles: Dependence upon Particle Size and Support. *ACS Catalysis* **2017**, *7* (12), 8460-8466. DOI: 10.1021/acscatal.7b03090.
(23) Ding, R.; Azadehranjbar, S.; Padilla Espinosa, I. M.; Martini, A.; Jacobs, T. D. B. Separating Geometric and Diffusive Contributions to the Surface Nucleation of Dislocations in Nanoparticles. *ACS Nano* **2024**, *18* (5), 4170-4179. DOI: 10.1021/acsnano.3c09026 From NLM PubMed-not-MEDLINE.
(24) Azadehranjbar, S.; Ding, R.; Padilla Espinosa, I. M.; Martini, A.; Jacobs, T. D. B. Size-Dependent Role of Surfaces in the Deformation of Platinum Nanoparticles. *ACS Nano* **2023**, *17* (9), 8133-8140. DOI: 10.1021/acsnano.2c11457 From NLM PubMed-not-MEDLINE.
(25) Zhang, L.; Cosandey, F.; Persaud, R.; Madey, T. E. Initial growth and morphology of thin Au films on TiO2(110). *Surface Science* **1999**, *439* (1-3), 73-85. DOI: 10.1016/s0039-6028(99)00734-7.
(26) Lazzari, R.; Renaud, G.; Jupille, J.; Leroy, F. Self-similarity during growth of theAu/TiO2(110)model catalyst as seen by the scattering of x-rays at grazing-angle incidence. *Physical Review B* **2007**, *76* (12). DOI: 10.1103/PhysRevB.76.125412.
(27) Giorgio, S.; Cabié, M.; Henry, C. R. Dynamic observations of Au catalysts by environmental electron microscopy. *Gold Bulletin* **2008**, *41* (2), 167-173. DOI: 10.1007/bf03216594.
(28) Hemmingson, S. L.; James, T. E.; Feeley, G. M.; Tilson, A. M.; Campbell, C. T. Adsorption and Adhesion of Au on Reduced CeO2(111) Surfaces at 300 and 100 K. *The Journal of Physical Chemistry C* **2016**, *120* (22), 12113-12124. DOI: 10.1021/acs.jpcc.6b03789.

(29) Montini, T.; Melchionna, M.; Monai, M.; Fornasiero, P. Fundamentals and Catalytic Applications of CeO2-Based Materials. *Chemical Reviews* **2016**, *116* (10), 5987-6041. DOI: 10.1021/acs.chemrev.5b00603.
(30) Yang, C.; Lu, Y.; Zhang, L.; Kong, Z.; Yang, T.; Tao, L.; Zou, Y.; Wang, S. Defect Engineering on CeO2-Based Catalysts for Heterogeneous Catalytic Applications. *Small Structures* **2021**, *2* (12). DOI: 10.1002/sstr.202100058.
(31) Farmer, J. A.; Campbell, C. T. Ceria Maintains Smaller Metal Catalyst Particles by Strong Metal-Support Bonding. *Science* **2010**, *329* (5994), 933-936. DOI: 10.1126/science.1191778.
(32) Bruix, A.; Rodriguez, J. A.; Ramírez, P. J.; Senanayake, S. D.; Evans, J.; Park, J. B.; Stacchiola, D.; Liu, P.; Hrbek, J.; Illas, F. A New Type of Strong Metal–Support Interaction and the Production of H2 through the Transformation of Water on Pt/CeO2(111) and Pt/CeOx/TiO2(110) Catalysts. *Journal of the American Chemical Society* **2012**, *134* (21), 8968-8974. DOI: 10.1021/ja302070k.
(33) Zhang, S.; Xia, Z.; Ni, T.; Zhang, Z.; Ma, Y.; Qu, Y. Strong electronic metal-support interaction of Pt/CeO2 enables efficient and selective hydrogenation of quinolines at room temperature. *Journal of Catalysis* **2018**, *359*, 101-111. DOI: 10.1016/j.jcat.2018.01.004.
(34) Peng, Y. F.; Guo, Y. B. An adhesion model for elastic-plastic fractal surfaces. *Journal of Applied Physics* **2007**, *102* (5). DOI: 10.1063/1.2777476.
(35) Zafeiratos, S.; Piccinin, S.; Teschner, D. Alloys in catalysis: phase separation and surface segregation phenomena in response to the reactive environment. *Catalysis Science & Technology* **2012**, *2* (9), 1787-1801.
(36) Liao, H.; Fisher, A.; Xu, Z. J. Surface Segregation in Bimetallic Nanoparticles: A Critical Issue in Electrocatalyst Engineering. *Small* **2015**, *11* (27), 3221-3246. DOI: 10.1002/smll.201403380.
(37) Fushiki, T. Estimation of prediction error by using K-fold cross-validation. *Statistics and Computing* **2009**, *21* (2), 137-146. DOI: 10.1007/s11222-009-9153-8.
(38) Garimella, R. V. *A simple introduction to moving least squares and local regression estimation*; Los Alamos National Laboratory (LANL), Los Alamos, NM (United States), 2017.
(39) Pacchioni, G. Electronic interactions and charge transfers of metal atoms and clusters on oxide surfaces. *Physical Chemistry Chemical Physics* **2013**, *15* (6). DOI: 10.1039/c2cp43731g.
(40) Chutia, A.; Willock, D. J.; Catlow, C. R. A. The electronic properties of Au clusters on CeO2 (110) surface with and without O-defects. *Faraday Discussions* **2018**, *208*, 123-145. DOI: 10.1039/c8fd00002f.
(41) Luo, Y.; Chen, Z.; Zhang, J.; Tang, Y.; Xu, Z.; Tang, D. Theoretical insights into ω-alkynylfuran cycloisomerisation catalyzed by Au/CeO2(111): the role of the CeO2(111) support. *RSC Advances* **2017**, *7* (22), 13473-13486. DOI: 10.1039/c6ra27207j.
(42) Tosoni, S.; Pacchioni, G. Trends in Adhesion Energies of Gold on MgO(100), Rutile TiO2(110), and CeO2(111) Surfaces: A Comparative DFT Study. *The Journal of Physical Chemistry C* **2017**, *121* (51), 28328-28338. DOI: 10.1021/acs.jpcc.7b09429.
(43) Liu, B.; Yan, Z.; Xu, T.; Wang, J.; Li, C.; Gao, R.; Bai, J. Promoting electron transfer of surface oxygen vacancies in Pd/CeO2-RE via doping engineering for enhancing catalytic activity in Suzuki coupling reaction. *Journal of Catalysis* **2021**, *399*, 15-23. DOI: 10.1016/j.jcat.2021.04.025.
(44) Burroughs, P.; Hamnett, A.; Orchard, A. F.; Thornton, G. Satellite structure in the X-ray photoelectron spectra of some binary and mixed oxides of lanthanum and cerium. *Journal of the Chemical Society, Dalton Transactions* **1976**, (17), 1686-1698.

(45) Bezkrovnyi, O.; Bruix, A.; Blaumeiser, D.; Piliai, L.; Schötz, S.; Bauer, T.; Khalakhan, I.; Skála, T.; Matvija, P.; Kraszkiewicz, P.; et al. Metal–Support Interaction and Charge Distribution in Ceria-Supported Au Particles Exposed to CO. *Chemistry of Materials* **2022**, *34* (17), 7916-7936. DOI: 10.1021/acs.chemmater.2c01659.
(46) Gao, J.; Luo, X.; Chang, W.; Wang, Z.; Yan, Y.; Geng, Y. Insight into Atomic-Scale Adhesion at the C–Cu Interface During the Initial Stage of Nanoindentation. *Nanomanufacturing and Metrology* **2022**, *5* (3), 250-258. DOI: 10.1007/s41871-022-00149-3.
(47) Wang, A.; Liu, X. Y.; Mou, C.-Y.; Zhang, T. Understanding the synergistic effects of gold bimetallic catalysts. *Journal of Catalysis* **2013**, *308*, 258-271. DOI: 10.1016/j.jcat.2013.08.023.
(48) Wei, X.; Yang, X.-F.; Wang, A.-Q.; Li, L.; Liu, X.-Y.; Zhang, T.; Mou, C.-Y.; Li, J. Bimetallic Au–Pd Alloy Catalysts for N2O Decomposition: Effects of Surface Structures on Catalytic Activity. *The Journal of Physical Chemistry C* **2012**, *116* (10), 6222-6232. DOI: 10.1021/jp210555s.
(49) Bond, G. C.; Louis, C.; Thompson, D. *Catalysis By Gold*; World Scientific Publishing Company, 2006.
(50) Doherty, R. P.; Krafft, J.-M.; Méthivier, C.; Casale, S.; Remita, H.; Louis, C.; Thomas, C. On the promoting effect of Au on CO oxidation kinetics of Au–Pt bimetallic nanoparticles supported on SiO2: An electronic effect? *Journal of Catalysis* **2012**, *287*, 102-113. DOI: 10.1016/j.jcat.2011.12.011.
(51) Mokkath, J. H.; Schwingenschlögl, U. Catalytically favorable surface patterns in Pt–Au nanoclusters. *RSC Advances* **2013**, *3* (35). DOI: 10.1039/c3ra42288g.
(52) Zeng, J.; Yang, J.; Lee, J. Y.; Zhou, W. Preparation of Carbon-Supported Core−Shell Au−Pt Nanoparticles for Methanol Oxidation Reaction: The Promotional Effect of the Au Core. *The Journal of Physical Chemistry B* **2006**, *110* (48), 24606-24611. DOI: 10.1021/jp0640979.
(53) Bus, E.; van Bokhoven, J. A. Electronic and Geometric Structures of Supported Platinum, Gold, and Platinum−Gold Catalysts. *The Journal of Physical Chemistry C* **2007**, *111* (27), 9761-9768. DOI: 10.1021/jp067414k.
(54) Wong, R. J.; Scott, J.; Kappen, P.; Low, G. K. C.; Hart, J. N.; Amal, R. Enhancing bimetallic synergy with light: the effect of UV light pre-treatment on catalytic oxygen activation by bimetallic Au–Pt nanoparticles on a TiO2support. *Catal. Sci. Technol.* **2017**, *7* (20), 4792-4805. DOI: 10.1039/c7cy01326d.
(55) Wang, D.; Cui, X.; Xiao, Q.; Hu, Y.; Wang, Z.; Yiu, Y. M.; Sham, T. K. Electronic behaviour of Au-Pt alloys and the 4f binding energy shift anomaly in Au bimetallics- X-ray spectroscopy studies. *AIP Advances* **2018**, *8* (6). DOI: 10.1063/1.5027251.
(56) Wu, P.; Liu, H.; Cao, Y.; Xi, S.; Li, Z.; He, Z.; Song, L.; Xu, J.; Bai, P.; Zhao, L.; et al. Mesostructured cellular foam silica supported Au–Pt nanoalloy: Enrichment of d-state electrons for promoting the catalytic synergy. *Microporous and Mesoporous Materials* **2021**, *316*. DOI: 10.1016/j.micromeso.2021.110982.
